# Fiftieth Anniversary of the Discovery of Anæsthesia by Horace Wells

**Published:** 1895-01

**Authors:** 


### Fiftieth Anniversary of the Discovery of Anæsthesia by Horace Wells.

Held in Philadelphia, December 11th, 1894.

(ABSTRACT).

Nearly one hundred visiting dentists, a large contingent of the local profession, and the students from the Dental Department, University of Pennsylvania, Philadelphia College of Dental Surgery, and from the Medico-Chirurgical College, to the number of eight hundred, assembled in Association Hall, at 2 P. M.

DR. J. D. THOMAS, Chairman of the Executive Committee, called the meeting to order, and introduced Dr. J. Y. Crawford, of Nashville, Tenn., President of the American Dental Association.

DR. CRAWFORD: Mr. Chairman, Fellows of the Dental Profession, Ladies and Gentlemen. At the meeting of the American Dental Association at Old Point Comfort, Virginia, on the second Tuesday of August last, a resolution to organize this memorial occasion was introduced by Dr. J. D. Thomas. This magnificent assemblage is the culmination of that resolution, and this is the gathering to celebrate the emancipation of the human family from pain by the discovery of anæsthesia. Fifty years ago to-day, Horace Wells made the first legitimate exhibition of anæsthesia, under proper restrictions, in a surgical way, which will entitle him to as much recognition at the hands of a just and liberal profession as was accorded to Jenner, Harvey, and Pasteur. The incident of the bruised knee was to Wells, what the inadvertant remark of the milk-maid was to Jenner. They were both pivotal contributions to the healing art!

DR. THOS. FILLEBROWN, of Boston, was introduced, and read an exhaustive paper on "The History of Anæsthesia." He said: Great discoveries and events do not burst forth with Promethian suddenness, but wait long periods of hope and may be despair. So it was with the discovery by Horace Wells, which robbed pain of its victory, and the knife of its horrors. The ancient nations hunted for anæsthesia. Homer mentions the inhalation of hemp for that purpose. Pliny and Apuleius refer to the mandragora wine; the Chinese used hemp in the third century; Theodoric described the *spongia somniferum* in the thirteenth century. Ether was also known at that time. In 1800, Sir Humphrey Davy said nitrous oxide might probably be used to advantage in surgical operations where there was no great effusion of blood. But the suggestion bore no fruit for nearly half a century. Of the two anæsthetic agents, Protoxide of Nitrogen and Sulphuric Ether, the former was discovered by Priestly, described by Davy, and applied by Wells; the latter was discovered in the thirteenth century, named in the eighteenth, and applied in the nineteenth.

In 1846, its use was made known by Dr. Wm. T. G. Morton at the Massachusetts General Hospital. The anæsthetic properties of nitrous oxide were long known and applied for the entertainment it afforded, but the chasm separating that from its true utility was unbridged until fifty years ago to-day, the man whose honor we celebrate, by inhaling the gas and having a tooth extracted without pain, fulfilled Davy's prophecy, and made practical anæsthesia a discovered and demonstrated reality. That event, the birth of Pain's Victor, was the source of our knowledge of anæsthesia, and made the name of Horace Wells echo around the world. In the following winter, Dr. Wells made a visit to Boston, and through the kindly offices of his former pupil and partner Dr. Morton, who afterwards introduced Ether, and was a claimant to the discovery of anæsthesia, made an exhibition to the Harvard medical class, by permission of Dr. J. C. Warren. The attenpt was an apparent failure. The patient was incompletely anæsthetized and cried out, as they frequently do, and Wells was greeted with hisses of derision. The patient afterwards said he experienced no pain. This failure so disheartened Wells, that he shortly abandoned the practice of dentistry and committed suicide by severing a jugular vein. With the unparalleled honor of the discovery by Horace Wells, the names of Dr. G. Q. Colton, Col. Samuel Cooley, Dr. J. M. Riggs, E. E. Marcy, W. T. G. Morton, Oliver Wendell Holmes, Jackson and Bigelow are indissolubly connected. Marcy suggested ether to Wells instead of nitrous oxide; Morton made the first public application of ether for surgical anæsthesia; Jackson claimed to have suggested ether to Morton, and Oliver Wendell Holmes suggested the name anæsthesia which is "repeated by the tongues of every civilized nation." Dr. Crawford W. Long, of Athens, Ga., claimed to have given ether three times in 1842–3. It was not printed until 1849. How could he resist flying with joyous wings to proclaim to a waiting world the great boon to humanity? The honor of the discovery is not accorded to Long, on account of its inauthenticity, and its tardy publication. If he used it, nobody knew it, and nobody used it because Long did. Then came the long list of pretenders, denominated generically "jump-up-behinders." In 1846, Sept. 30, Dr. Wm. T. G. Morton administered sulphuric ether for the first time. He made a public demonstration at the clinic of Dr. John C. Warren, at the Massachusetts General Hospital on Oct. 16, 1846. Morton and Jackson made a joint oath to the discovery of ether as an anæsthetic and applied for a patent: the next year each claimed to be the individual discoverer. The application was discarded and declared not patentable. In 1847, the Paris Academy proclaimed Morton and Jackson the joint discoverers, but after a full hearing, they decided that the honor belonged to Horace Wells, as the first to use gases and vapors to perform surgical operations without pain. In 1847, Sir James Y. Simpson of Edinburgh made experiments which gave chloroform as an anæsthetic to the world. Whom, then, shall the honor of anæsthesia's discovery make immortal? To each and all of the glorious names who made their individual contributions; but the noble, generous mind, that conceived the grand idea, and conferred the greatest boon of science on humanity belonged to the immortal spirit of Horace Wells. The facts maintain the truth of his priority; disputation but weakens the evident conclusion. In honoring his memory, we should regret his sad and tragic end. It is a peculiar fact that his rival claimants both met violent deaths. Morton died suddenly of apoplexy, while Jackson was insane the last seven years of his life. Thus did the Shears of Fate cut the tent-ropes of their lives. Let us lay the chaplet of honor on his memory. Would we might with it crown his head. The everlasting epitaph of this martyr and hero will be,

"TO THE DISCOVERER OF ANÆSTHESIA—HORACE WELLS!"

Prof. James E. Garretson, of Philadelphia, was introduced. After thanking the audience for their ovation, he said there was in the audience one whose shoe-latchets he was unworthy to tie—Dr. G. Q. Colton. We are here to honor Wells. Without a Colton, there could be no Wells. We are going to erect a statue to Wells. Let us begin by erecting a human monument—Dr. G. Q. Colton. Dr. Colton was presented and seated upon the stage. Prof. Garretson made an address on "The Benefits of Anæsthesia to Mankind." He said he was overwhelmed at the contrast of the occasion and the speaker. It is not profanation to compare the reverence of a priest, when he uncovers the Host, in the profundity of holiness, to his own feelings when he speaks of this greatest of God's gifts to humanity "Silence is Golden." Anæsthesia is the gold of silence. The silence of pitying lips in the presence of torture, shorn of its horrors. The ring of a bell is in its metal; the ring of a man is in his work. Horace Wells! It does not nor will not still. It rings and rings and rings in distinctness, albeit accordant and discordant sounds are everywhere around. He was a vessel capable of holding and was filled. In him the river of Lethe found a channel. Everywhere over the land flows the stream of Nepenthe. The melody of music is not a note; the inspiration of a poet is not grammer. The ghost of anæsthesia was in the camel droppings on the desert, in the fields red with poppy. Ether was known to Frobenius. But who dreamed of the wonderland of Ruthenasia, contained in the bottle on the chemist's shelf? Cadmius saw letters. Shakespeare saw the fulness of expression. Horace Wells saw in the room at Hartford, what had ne'er been seen before—Anæsthesia. Some had seen the filmy halo that meant anæsthesia, but it was forgotten. Sir Humphrey Davy saw the outskirts of Elysium; but it was only in thought. The seership of Horace Wells was practical. While pain is painful, his name will be upon the lips of men. Apples ripened and fell before Davy guessed their secret; kettles boiled and hissed without telling their story; electricity flashed athwart the firmament long before it was harnessed; the sun's rays made perfect pictures; but there were no takers of these gifts. Alexander told the story of steam; Pipon invented the cylinder; Fulton launched a steamboat, and Stevenson a train of cars; Daguerre made beautiful counterfeits of nature by the aid of the sun; and Mozart told by note what the flowers were doing. Was anæsthesia as anæsthesia known to surgery before 1844, as it became known in that year and has been known since? Not nitrous oxide, or ether, or chloroform; not rapid breathing but anæsthesia? The man of that year was Horace Wells. Anæsthesia! What would the world do without it! What could it do! What did it do! Think of an operation without it. A mother with tear bedimmed eyes, in despair and misery follows with trembling steps, the nurse who bears her first born to the operating table. The cries of the innocent babe mingle with the agonizing shrieks of its mother. It is held by force; she is torn from its side, and as she hears the heart-rending moan, falls down in a heap and is borne from the room screaming and crazed, cursing God as a being without mercy. Now, a child who has a deformity to be corrected, is cuddled while he crowingly inhales the subtle fumes of chloroform, and dreams of babyland embowered in roses while the operation is quickly accomplished. The name of the maker of this picture? Horace Wells! Hail to the Poets, Musicians, Seers, whose statues of enduring brass mark our working places! Hail to all the Seers! Immortals! Hail to Horace Wells!

The Chairman announced that the consideration of a plan for a permanent memorial to the discovery by Horace Wells would be entertained.

Dr. L. D. Sheppard offered some resolutions drafted by the Executive Committee, in reference to the discovery of Anæsthesia, which were approved with unanimity.

Dr. R. Huey of New York moved that a Committee be appointed by the President of the American Dental Association, to secure funds for the erection of a Memorial in Washington City.

Approved. (Committee to be announced).

The chairman introduced Dr. G. Q. Colton, of New York, who gave the following historical reminiscence:

In the words of Anthony at the funeral of Cæsar, I can say:

"I am no orator, as Brutus is;
But, as you know me all, a plain blunt man,
For I have neither wit, nor words, nor worth,
Action, nor utterance, nor the power of speech
To stir men's blood: I only speak right on;
I tell you that which you yourselves do know."

On the 10th of December, 1844, I gave an exhibition of the amusing effects of the nitrous oxide gas, in the city of Hartford, Conn. After a brief lecture on the properties and effects of the gas, I invited a dozen or fifteen gentlemen to come upon the stage who would like to inhale it. Among those who came forward was Dr. Horace Wells, and a gentleman by the name of Cooley. Among those who inhaled the gas was Mr. Cooley. When under its influence, he began to dance and jump about. He ran against some wooden settees on the stage and bruised his shins badly. When recovering from the effects of the gas, he went to his seat, next to Dr. Wells. Dr. Wells said to him: "You must have hurt yourself." "No," said Cooley, but at the same time he began to feel some pain in his legs. He was astonished to find his legs all bloody, said he felt no pain till the effects of the gas had passed off. At the close of the exhibition, and while the audience was retiring, Dr. Wells came to me and said: "Why cannot a man have a tooth extracted when under the influence of the gas, and not feel it?" I replied that I did not know, as the thought had never entered my head. Dr. Wells said he believed it could be done; and that if I would bring a bag of the gas to his office the next day, he would try it himself. The next day I took a bag of the gas to his office, and Dr. Wells called in Dr. Riggs, a neighboring dentist, to perform the operation. I administered the gas to Dr. Wells, and Dr. Riggs extracted a decayed molar tooth. On recovering and finding his tooth out, Dr. Wells slapped his hands upon his knee and exclaimed, very excitedly, "*It is the greatest discovery ever made. I didn't feel it so much as the prick of a pin!*" That was the first tooth ever drawn without pain, and was the birth of anæsthesia. This operation took place just fifty years ago today. The discovery of anæsthesia and its practical demonstration belongs entirely to Dr. Wells.

MR. CHARLES T. WELLS, of Hartford, Conn., the only son of the great discoverer was presented to the audience.

DR. DONNELLY, of Washington, moved that the committee be instructed to take into consideration the feasibility of establishing a National Memorial Hall in connection with the Wells' monument.

BANQUET.

A magnificent banquet was held at the Union League, at 6:30 p. m.

DR. E. T. DARBY, of Philadelphia, presided as Toast-Master. General Joseph B. Hawley, U. S. Senator from Connecticut, responded to the toast, "The Horace Wells' Discovery—Its National Significance." He asked: How many thousand years were added to human life by the result of the great discovery? How many years of agony were thrown into the bottomless pit of oblivion? He knew the office in which this discovery was made. He knew the gay and frisky Col. Sam Cooley who danced about and barked his shins, and was the innocent cause of the brilliant discovery. He exhibited the book of Wells. Truman Smith, the venerable lawyer of Connecticut, and scores of Hartford's great men testified to the validity of Well's claim. He had been present the day before at the anniversary celebration in Hartford. It took those eighty-year old enthusiasts until midnight to erect the bronze tablet. He felt honored at being present at both celebrations.

PROF. JAMES TRUMAN, of the University of Pennsylvania, responded to the toast: "Anæsthesia as a Dental Discovery." Prof. Truman was reminded of the story of the Ugly Duckling that came out late, was picked at, and loved by no one. But it was able to swim and fly, and was adopted by a tribe of wild ducks, and afterwards became a beautiful swallow. Dentistry came in late—the last half century; but to-day her representatives have assembled here from fourteen states, in a high professional spirit, to do honor to one of her greatest men. Horace Wells lived in the period of transition in dentistry, when every man's hand was against his neighbor in professional matters. He was broader. He reached out after the great world that Goethe loved. He went to the centre of medical education—Boston—and was hooted out of the medical presence in disgrace. But every age has stoned her prophets, as every age will continue to do. Dr. B. W. Richardson, of London, in the last few weeks, in Longman's Magazine has tried to tear the laurel from Horace Wells and place it on Sir Humphrey Davy. The parable of the sower is applicable. Priestly was the stony ground; Sir Humphrey Davy was the poor soil—he was a dreamer. The receptive brain of Horace Wells was the good soil that bore fruit in the amelioration of pain. When for the first time modern anæsthesia was exhibited, amid the anxiety of the surgeon, the excitement of the students, when for the first time they beheld a patient passive under the surgeon's knife, did anybody think of Sir Humphrey Davy? When we look back over the great battles, the terrors of hospitals and the accidents of life, who can aggregate the benefits of anæsthesia? It was in the humble home of the Hartford Dentist that the still small voice whispered in the wilderness of suffering. And that whisper will echo and re-echo until the cry of agony shall be silenced forever. O Dentistry! though not the first born of this our nineteenth century, in our heart of hearts we enshrine thee. Thou hast given anæsthesia to the world.

Prof. J. William White, of the University of Pennsylvania, responded to the toast, "Anæsthesia as a Factor in the Evolution of Surgery." The discovery of anæsthesia is a priceless gift to surgery. Like an enchanted Genii of the Arabian nights it transports one from conscious suffering to the dreamy slumber of oblivion. If it had contributed nothing more to the victories of surgery than the transformation of a screaming sufferer into a plastic, unconscious patient for the surgeon's knife, it had added incalculably to its efficiency. But that is the least of its blessings. It brought possibilities of an incredulous advance. Hundreds of operations undreamt of in 1844 have saved the lives of countless thousands. The processes of disease and trauma in regions uninvaded in pre-anæsthetic days, were helpless and hopeless before the inspiration of Wells fifty years ago. The advance of this period has outstripped that of eight hundred years. In it, aseptic and antiseptic surgery has developed, and almost wiped out certain forms of suffering, disease and death. Surgery has not reached its culmination. Investigation, research and experiment are advancing rapidly. The prizes are still great; tubercle and cancer remain to be conquered, and though we may not live to see it, it will ultimately be accomplished. The age is full of glorious men vigorously anticipating the splendid hopes of the future. All glory to Horace Wells, the builder of the foundation and the layer of the corner stone.

PROF. HORATIO C. WOOD, of the University of Pennsylvania, responded to the toast: "The Debt of Medicine to Anæsthesia." Once there were twins. One was lusty and eager, always shouting its own praises; at the fore front of battle, revelling in blood, accident and death. The other was modest and retiring, thinking much but speaking little. And one was Surgery and the other Medicine. To the twin Surgery, anæsthesia came as a great gift. To Medicine, it did'nt at first appear to be such a great boon. But there are now many diseases that attack the mortal frame, that could not be relieved without the great gift from Hartford. Were it not for anæsthesia, few would have the courage for vivisection; and were it not for vivisection, there were no modern medicine. Anæsthesia has made modern physiology, antiseptic surgery and advanced medicine the great wonderful structure that we stand off and contemplate with such reverential awe. That is what anæsthesia has done. Not simply to quell pain momentarily, but made possible modern medicine. He hoped the dental profession would erect a monument to one of their guild who was such a benefactor to mankind. He did not know of a single statue erected to a medical man in the United States. When Leidy died, the greatest man Philadelphia ever produced, the one man who was ever crowned by the immortal leaf of the French Academy, the newspapers only had five or six lines about him.

Col. Alexander McClure, Editor of *The Times*, Philadelphia, responded to the toast, "The Mastery of Pain from the Standpoint of the Layman." Col. McClure remembers the transition of dentistry from a critical condition to its present position of wonderful achievement. He remembered when the blacksmith of the village pulled teeth with a gimlet having a screw in its end, and the itinerant dentist "stood" one day at one place and the next at another. He recollected the introduction of anæsthesia into the faculty forty-five years ago. He was then publishing a country newspaper—the only time in his life when he thoroughly understood the newspaper business. He and his apprentice instituted a series of experiments. The apprentice proposed to have a young physician give him chloroform, the Colonel was quite willing that it should be. It was administered with great success. Every case of type being upset. He claimed some credit for its advance. About thirty years ago he was suffering with an aching wisdom tooth. He had read of the gas. He came to Dr. Thomas, saw the list of some eighteen hundred persons who had taken it successfully; but he saw no instruments. He took a deep inhalation—then another—then . . . . ! Suddenly he woke up, and asked if the tooth had been pulled. He was assured by seeing the offending member. Since then, when he has a tooth to be extracted, he quits work at midday, walks quietly to the dentists' office, takes a whiff of gas, and all is over. Dr. White tried to cut him to pieces seven times, but he was ignorant of the pain. He was satisfied that his life had been saved by the operation, and but for anæsthesia, he would not have been present.

Prof. Wilbur F. Litch, of the Pennsylvania College of Dental Surgery, responded to the toast, "The Development of our Knowledge of Anæsthesia." He said the discoverer of anæsthesia had done more to promote the happiness of mankind than all the philosophers from Sophocles to Mill. In 1832, Velpean declared painless operations in surgery a chimera; but. later, he performed an amputation under ether. The ancients sought for some analgesic in mendragora, hyoscyamus, opium and hemp. The stupefying effects of alcohol were more safe and effective. The marvel is why alcohol to its full intoxicating effect was not systematically employed. "Years teach much which days never know." (Emerson). Ether was known five hundred years; nitrous oxide seventy years before Wells' time. Anæsthesia is a flower that has blossomed slowly on the cross of suffering. It is recorded that the Romans offered a lethal draft to Him who bore the typification of human suffering. Humanity today drinks of subtler influences. He referred to the report of the Hyderabad commission on chloroform, and mentioned the use of cocaine in the production of local anæsthesia. Ideal anæsthesia, one that was perfectly free from danger, remained to be discovered. At present, nitrous oxide with oxygen is the best, but for mechanical difficulties.

District Attorney GEO. S. GRAHAM, of Philadelphia, responded to the toast, "The Medico-legal Aspect of Anæsthesia." The toast reminded him of Daniel O'Connell calling a man a nefarious ruffian because the phrase was high-sounding. The story of a man knocking a hole in a cellar wall to let the dark out was applicable to it. Anæsthesia in its broad sense including alcohol, made many subjects for the legal surgeon's knife. When Dr. Thomas gave him gas for the extraction of a tooth, he heard a seraphic symphony from the heavenly spheres, but when he awoke, he found the music came from a music-box, and was administered with malice aforethought. That was the first connection of anæsthesia with the law. He said law was not in sympathy with vivisection. He once lost a dog himself. The highest tribute from a sister profession is the reiteration of the praise bestowed by one's own profession. If a man lives in their memory, and is honored as the discoverer of a great good to suffering humanity, that is the loftiest pedestal on the footstool of God. Esteem and honor to pioneers in discovery and advancement. The whole world joins in sweet acclaim of praise to the memory of Horace Wells.

REV. S. D. MCCONNELL, of Philadelphia, responded to the toast, "The Humanitarian Aspect of Anæsthesia." Most parsons have learned the ability to escape from the text; but he would rather be the discoverer of anæsthesia than to be any man that ever lived. When all the achievements of this century shall be forgotten, this one great controlling event that happened once in the history of humanity will remain. The old books on theology, discussed at interminable length the meaning and use of pain, claiming that it was eternal, insoluble, the result of evil, and the punishment therefor. The measure of sensibility to pain is the measure of civilization. Low civilization is comparatively indifferent to pain. This is an age of physical anæsthesia; of moral and mental æsthetics. All the philosophizing about pain cannot make us bear with equanimity some other fellow's pain. Pain is demoralizing, and its relief is elevating. It begets gentling of manners and thought, tenderness and compassion. The man who has done this, has taken out part of the unspeakable anguish of parturition, has saved innumerable lives, has enabled timid souls to look serenely upon suffering, and walk triumphantly to the end.

Dr. G. Q. Colton related some amusing experiences in connection with the administration of nitrous oxide and tooth pulling, and concluded with the masterly advice of Polonius to his son Laertes.

Mr. Charles Wells, the only son of the immortal dentist, gave some personal recollections of his father.

Adjournment.

